# Effects of Integrating Pain Coping Strategies into Occupational Therapy After Total Knee Arthroplasty: A Parallel Mixed-Method Study

**DOI:** 10.3390/healthcare13060627

**Published:** 2025-03-13

**Authors:** Ryusei Hara, Yuki Hiraga, Yoshiyuki Hirakawa, Akira Babazono

**Affiliations:** 1 Fukuoka Rehabilitation Hospital, Fukuoka 819-8551, Japan; ryusei132@gmail.com (R.H.); yutsuki0903@yahoo.co.jp (Y.H.); 2 Department of Occupational Therapy, Faculty of Medical Science, Fukuoka International University of Health and Welfare, Fukuoka 814-0001, Japan; 3 Department of Health Care Administration and Management, Graduate School of Kyushu University, Fukuoka 812-0054, Japan; babazono@hcam.med.kyushu-u.ac.jp

**Keywords:** total knee arthroplasty, pain coping, Canadian Occupational Performance Measure

## Abstract

**Background/Objectives**: This study aimed to evaluate whether integrating coping strategies into occupational therapy (OT) enhances functional recovery and psychological adaptation after total knee arthroplasty (TKA). **Methods**: Twenty-eight patients who underwent TKA were equally assigned to an intervention and control group. Both groups received standard goal-oriented OT, while only the intervention group underwent structured training in pain coping strategies using the “Coping List”. Treatment effects were assessed using Canadian Occupational Performance Measure (COPM), pain, anxiety, depression, and pain-related disability scores. **Results**: A total of 210 coping strategies were identified, with the intervention group adopting an average of 15.1 additional strategies per patient. Strategies were categorized into six domains: physical, psychological and cognitive, social support, relaxation, daily activities, and medication management. The intervention group showed significantly greater improvements in COPM performance scores (7.6 ± 1.7 vs. 5.5 ± 2.6; *p* = 0.048) and COPM satisfaction scores (7.9 ± 2.0 vs. 5.6 ± 2.8; *p* = 0.049) compared to the control group. **Conclusions**: The findings suggest that individualized coping strategies integrated with physical rehabilitation can help patients achieve postoperative goals, enhance recovery, and improve overall well-being. Incorporating such strategies into OT appears to be effective in early-postoperative rehabilitation. **Clinical trial number:** This study’s clinical trial registration information is available online at UMIN (UMIN000050536).

## 1. Introduction

Total knee arthroplasty (TKA) is a surgical intervention renowned for its capacity to alleviate pain and improve physical function and overall quality of life (QOL) in patients with knee osteoarthritis [1]. Outstanding postoperative outcomes have been documented, with satisfaction rates exceeding 90% at the five-year mark [2]. However, a significant subset of patients (10–34%) continues to experience moderate to severe pain for an extended period of more than three months following the procedure [3]. Psychological factors, including depression and catastrophic thinking, have been identified as contributing to chronic pain in individuals who have undergone TKA [4]. Furthermore, according to the fear-avoidance model, once individuals experience pain, their adverse perception of the discomfort can engender catastrophic thinking and avoidance of painful stimuli, thereby establishing a detrimental cycle of depression, physical inactivity, and disruptions in daily functioning [5]. Early-postoperative pain may also mediate psychological factors and lead to feelings of helplessness in the early-postoperative period [6]. Additionally, the course of pain in the first postoperative six months influences the long-term prognosis, suggesting the importance of rehabilitation during this period [7].

In recent years, cognitive behavioral therapy (CBT) has been identified as an effective intervention in patients with chronic pain [8]. Furthermore, the early use of CBT after TKA reduces pain during activity and catastrophic thinking, which is a key factor in chronic pain [9]. Despite the proven clinical effects of early-postoperative CBT, the presence of clinical psychologists in orthopedic centers remains limited, creating a gap in the care delivery system.

Against this background, the potential role of occupational therapy (OT) has been acknowledged, and the efficacy of OT interventions [10] and goal attainment [11,12] has been reported for postoperative patients with knee osteoarthritis, including those who have undergone TKA and high tibial osteotomies. Furthermore, as a specific intervention strategy, self-management through pacing techniques using an activity diary [13,14] has been found to improve pain control, physical function, and psychological factors, thereby establishing a clinical foundation for early-postoperative pain management in Japanese OT practices. Owing to the scarcity of reports on interventions incorporating coping techniques, which involve conventional CBT techniques used in OT for patients who are in the early-postoperative period after TKA, a case series was conducted to assess their effects [14]. Moreover, coping with pain entails making changes in daily life and activities [15]. Thus, self-involvement is essential and influenced by the following factors: age; physical, psychological, and social factors; and knowledge of medical conditions and coping strategies. An individual’s chosen coping behaviors can have both positive and negative outcomes [16]. Therefore, to ameliorate pain, it is crucial to encourage self-involvement and provide support in developing coping strategies involving multiple factors. Positive outcomes were observed in the case series, including improved Canadian Occupational Performance Measure (COPM) scores, pain levels, and psychological factors. However, owing to the nature of the report as a case series utilizing coping strategies in conjunction with OT practice, a comprehensive validation of the study’s efficacy could not be conducted.

Hence, this study aims to evaluate the effects of OT incorporating structured pain coping strategies on occupational performance and satisfaction, pain levels, anxiety, depression, quality of life, and pain-related disability in postoperative patients following knee replacement surgery. Additionally, we aim to analyze the characteristics of the coping strategies used during the early-postoperative phase and explore appropriate approaches to optimize rehabilitation outcomes.

## 2. Methods

### 2.1. Parallel Mixed-Method Design

We utilized a parallel mixed-method design to comprehensively evaluate the effectiveness of OT interventions for patients who had undergone TKA. Patients were divided into two groups: (1) an intervention group that followed the standard rehabilitation protocol supplemented with OT interventions, including coping strategy suggestions, and (2) a control group that adhered to the standard rehabilitation protocol without the coping strategy component. To evaluate the intervention’s effectiveness, both qualitative and quantitative data were collected. Qualitative data were obtained from coping lists documented during individual interviews, capturing effective coping strategies employed by patients in the early-postoperative period. These data were analyzed to explore strategy application in the intervention group. Quantitative data were gathered using validated outcome measures, including the COPM, the Numeric Rating Scale (NRS), and the EuroQol 5-Dimension 5-Level (EQ-5D-5L), and analyzed to assess changes in performance, satisfaction, pain, and QOL before and after the intervention. This mixed-method approach provided a comprehensive understanding of the impact of OT interventions incorporating coping strategies on patients’ recovery.

### 2.2. Patients

#### 2.2.1. Sample Size Estimation

A pre-test power analysis was conducted using G*Power [17] to estimate the sample size. The analysis was conducted to detect a medium effect size (Cohen’s *d* = 0.7) for primary and secondary outcomes. These outcomes included COPM performance and satisfaction scores, as well as measures of pain, anxiety, depression, pain-related lifestyle disability, and QOL. All parameters were measured pre and post intervention. Interactions between time (pre and post intervention) and group (intervention and control) were analyzed to evaluate the effectiveness of OT interventions with coping strategies. An estimated enrollment of 24 patients (12 in each group) was established to achieve a power of 0.5, the lowest level, and a significance (α) of 0.05.

#### 2.2.2. Patient Selection

**Figure 1:**
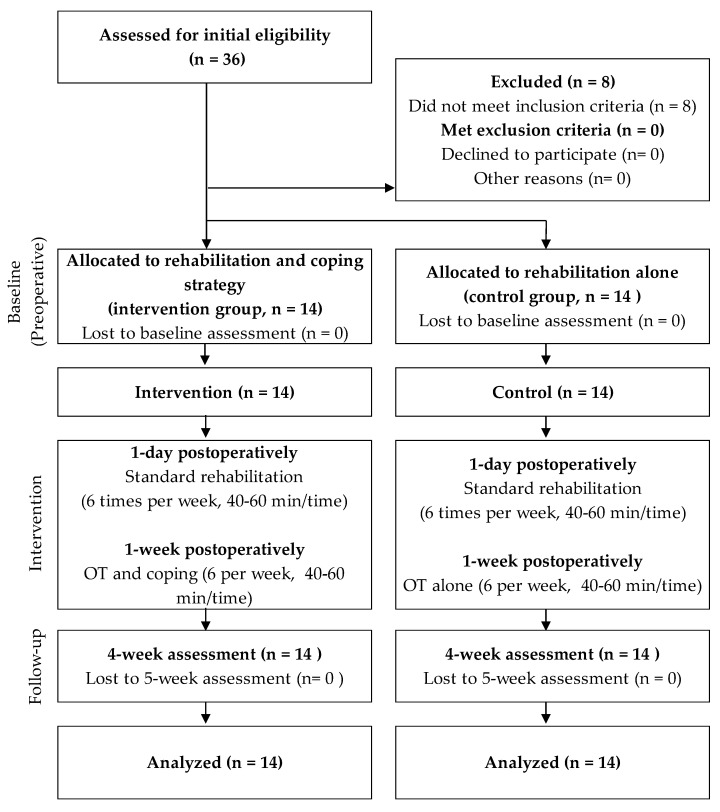
Study protocol.

The study population consisted of 36 women aged over 60 years who had undergone unilateral TKA at the Fukuoka Rehabilitation Hospital and had been diagnosed with knee osteoarthritis. Of these, 28 patients were included in this study after excluding those who (1) had psychiatric or neurological conditions, (2) self-reported a diagnosis of inflammatory arthritis, or (3) were scheduled for high tibial osteotomy for conditions other than knee osteoarthritis (Figure 1). Fourteen patients were assigned to the control group that received OT without coping strategies from January to December 2021, while the remaining fourteen were assigned to the intervention group which received OT with coping strategies from January to December 2022.

### 2.3. Outcomes

Each metric was evaluated both at baseline and at follow-up.

#### 2.3.1. Primary Outcomes

For goal-setting, the OT sessions were conducted using the COPM [18], which was validated by McColl et al. [19] for its utility in the community setting. The top five patient goals were recorded and prioritized according to their importance. The achievement and satisfaction for each goal were then rated using a 10-item scale, and the mean values for each item were calculated. The COPM has been translated and used in many countries and has a middle-to-high degree of reliability (intraclass correlation coefficient = 0.30 to 0.70) [20]. The correlations between the changes in the scores ranged from 0.42 to 0.66 for the Japanese version of the COPM [21].

#### 2.3.2. Secondary Outcomes

Pain was assessed using the NRS, rated on an 11-point scale from 0 (no pain) to 10 (excruciating pain) [22]. The NRS has good internal consistency (Cronbach’s α = 0.86) [23].

The Hospital Anxiety and Depression Scale (HADS) was used to assess anxiety and depression levels [24]. This self-administered questionnaire comprises fourteen questions divided into two scales—anxiety and depression—with seven questions each. Furthermore, each question is scored from 0 to 3, with higher scores indicating greater symptom severity. The scores for each scale are summed separately to provide total scores for anxiety and depression. The total scores for each scale range from 0 to 21, with scores of 0 to 7 indicating no anxiety or depression, scores of 8 to 10 suggesting suspected anxiety or depression, and scores of 11 or higher indicating confirmed anxiety or depression. The HADS has good internal consistency (Cronbach’s α = 0.80) [25].

The Pain Disability Assessment Scale (PDAS) was used to assess pain-related life disability [26]. The PDAS is a self-administered questionnaire developed in Japan to assess pain-related disability in patients with chronic pain. The scale comprises 20 items, each scored from 0 to 3, with higher scores indicating greater disability and a cutoff value of 10. The scale is based on the Sickness Impact Profile developed by Bergner et al. [27] and the Health Assessment Questionnaire developed by Fries [28]. The instrument was developed as a preliminary 32-item scale focusing on physical exercise and mobility. After rigorous item analysis, the scale was refined to 20 items. The PDAS has good internal consistency (Cronbach’s α  =  0.87 to 0.95) [26].

The EQ-5D-5L questionnaire was used to assess QOL. It includes five dimensions (mobility, self-management, usual activities, pain/discomfort, and anxiety/depression), with five response options for each dimension. Total scores range from 0 to 1, with higher scores indicating a better QOL. Regarding reliability, an internal consistency coefficient, specifically an intraclass correlation coefficient, of 0.75 has been reported for post-orthopedic patients [29]. Regarding validity, the EQ-5D-5L index demonstrates a moderate-to-strong correlation (ρ = −0.45 to −0.76) with the five items of the previous version, the EQ-5D-3L [30]. These results indicate that the EQ-5D-5L is a reliable and valid tool to assess health-related QOL.

### 2.4. Intervention

#### 2.4.1. OT Using Coping Strategies

**Figure 2:**
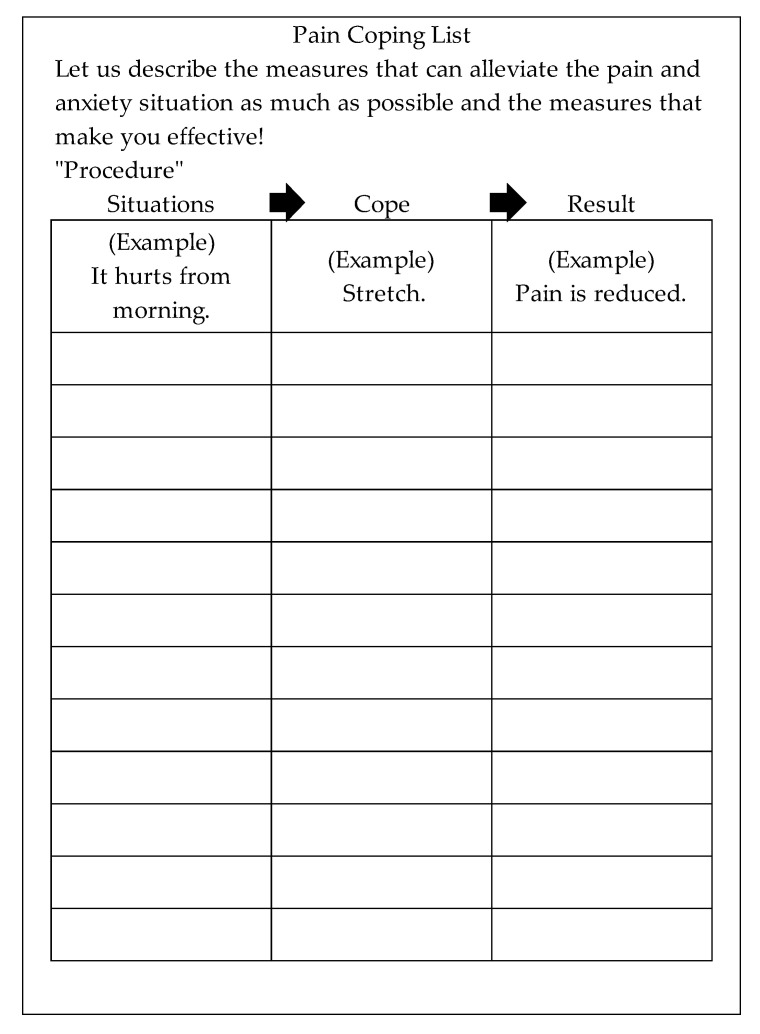
Coping list. *Note:* The strategy is to describe how to deal with pain and anxiety in daily life and what kind of results are obtained.

In addition to the regular rehabilitation program, the intervention group used a “coping list” (Figure 2) for coping strategy acquisition [15]. This tool helps develop coping strategies for dealing with pain and anxiety and examines how strategies used in these situations influence the development of new ones. This creates a feedback loop to assess the effectiveness of coping strategies and select appropriate ones. The specific intervention steps are outlined below.

STEP 1: Goal-Setting (First Day of OT Intervention): Patients were interviewed using the COPM to establish goals.

STEP 2: Gather Information on Current Pain Status and Coping Strategies (First Day of OT Intervention): After goal-setting, patients were interviewed about their current pain status. Their daily routine at the time of admission was documented, along with information on their activity, pain, and anxiety levels. Patients were then asked about the coping strategies they were already using. As many patients had limited knowledge of coping strategies, the concept was explained as efforts to manage pain and anxiety. Interventions to encourage the development of additional strategies were also suggested.

STEP 3: Coping List (First Day of OT Intervention): With the patients’ consent, a coping list (Figure 2) was provided to assist them in developing coping strategies. The list was explained, and examples from real-life pain and anxiety situations experienced the previous day were used to aid comprehension if needed.

STEP 4: Feedback and Reinforcement of Practice (From the Day After OT Intervention): Feedback was provided based on the results of practiced strategies to reinforce the use of the coping list. Patients unable to implement active coping were encouraged to reflect on their activities and thoughts during pain-free moments and practice strategies during painful or anxious episodes. Suggestions included diversifying coping strategies (e.g., expanding “listening to music” to include different genres) and increasing the number of available strategies. For those struggling, a list of common activities and thoughts was provided.

STEP 5: Self-Administration and Practice (From the Day After OT Intervention to the Day of Discharge): Patients practiced coping strategies to self-manage their pain and anxiety as part of a daily routine until discharge. This process was incorporated as homework during hospitalization. Feedback was provided only as necessary, and the interventions aimed to increase the number of effective coping strategies for each patient. After the study was concluded, data from the coping strategy lists were analyzed using the Jiro Kawakita (KJ) method [31].

#### 2.4.2. Usual Rehabilitation

All patients underwent TKA under general anesthesia, followed by a standard postoperative rehabilitation program. This program included knee-specific range-of-motion training and cooling therapy initiated on the first postoperative day. Patients began ambulation with a walker during the first week and worked on goal-oriented OT based on COPM goals from the second week [12,18]. Both physical therapy and OT were performed six times per week, dedicating 40–60 min per day for each session. Most patients began walking with a cane by the third week. A final evaluation was performed in the fourth week, after which the patient was discharged.

### 2.5. Confounding Variables: Participant Characteristics

Age, sex, body mass index, employment status (preoperative), smoking and drinking habits, and long-term care insurance information were obtained from Fukuoka Rehabilitation Hospital’s electronic medical records. In 2000, a new public long-term care insurance system was launched in Japan, based on which long-term care insurance became available to patients who required support (levels 1 and 2) and long-term care (levels 1 to 5). Long-term care certification is classified into seven levels: support 1 to 2 and long-term care 1 (the least disabled) to long-term care 5 (the most severely disabled). In this study, the system was used to classify patients’ illness severity in the control and intervention groups. This enabled the researchers to evaluate and compare the patients’ level of care and independence.

### 2.6. Qualitative Data Collection

Qualitative data were collected from the intervention group to explore the effective coping strategies used in the early-postoperative period. The data included strategies that were deemed effective and chosen from a list provided by the 14 participants in the intervention group. As mentioned previously, these coping strategies were documented using a coping strategy list. In their daily interviews, participants were asked about the effectiveness of the strategies they used the previous day. This collaborative process allowed the researchers to gather detailed information on which strategies were most effective and how they were implemented.

Interview Guide

What kind of pain or anxiety did you experience?

What coping strategies were considered in response to that situation?

Were the coping strategies you implemented effective? Why or why not?

### 2.7. Qualitative Analysis

Qualitative analysis was conducted using the KJ method, known for its ability to organize and visualize complex data, particularly large volumes of diverse information [31]. The analysis was performed jointly by the first and second authors, with guidance from the second author, who has extensive experience in qualitative research. The process began by labeling coping strategy data collected from the intervention group and organizing them under corresponding labels based on patient feedback about the effectiveness of the coping strategies. The data were then categorized based on similarities, including a visual exploration of the relationships among categories by placing related cards in close proximity and arranging them spatially. This step highlighted the similarities and differences among categories, facilitating an understanding of how each coping strategy related to others. The KJ method facilitated the grouping of the final dataset and helped draw meaningful conclusions from the study.

### 2.8. Statistical Analysis

Before comparing the baseline characteristics of the control and intervention groups, the normality of all data was assessed using the Shapiro–Wilk test. Depending on variance homogeneity, either Student’s *t*-test or Welch’s *t*-test was applied for continuous variables, while chi-squared (χ^2^) tests were used for categorical variables. To evaluate changes in COPM performance and satisfaction scores, as well as NRS, HADS, PDAS, and EQ-5D-5L scores, a repeated-measures two-way analysis of variance was conducted. This analysis included two factors, time (pre and post intervention) and group (control vs. intervention), with the significance level set at 0.05. Given the expected interaction between time and group, a repeated-measure analysis was performed while considering the four conditions: pre and post intervention within both the control and intervention groups. For variables showing significant interaction effects, post hoc multiple comparisons were performed using the Tukey–Kramer method. Additionally, effect sizes were calculated using Cohen’s *d* and interpreted according to the thresholds proposed by Livingstone et al. [32]: small (0.20 to 0.49), medium (0.50 to 0.79), and large (≥0.80). All statistical analyses were performed using JMP 14.0.

## 3. Results

### 3.1. Participant Characteristics

All 28 participants completed the study. Their demographic characteristics are exhibited in Table 1. The mean age of the participants was 76.8 ± 6.0 years. The mean age of the control group was 75.7 ± 5.7 years, and that of the intervention group was 77.9 ± 6.0 years.

### 3.2. Changes in Outcome Measure Indicators

**Figure 3:**
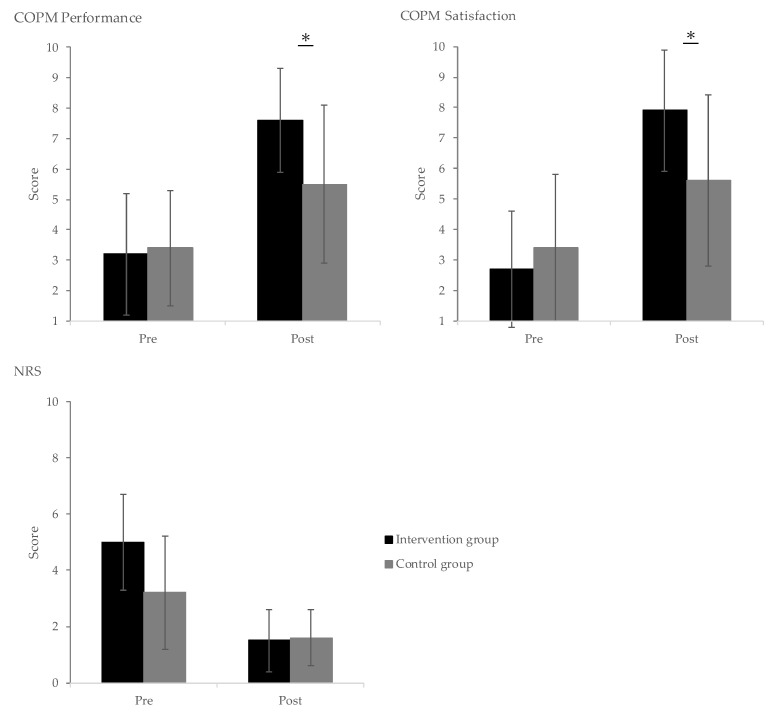
Multiple comparisons of intervention and control groups. *Note.* COPM, Canadian Occupational Performance Measure; NRS, Numerical Rating Scale; and CI, confidence interval. (*) A significant difference between groups was observed in the post-intervention results at two weeks (*p* < 0.05).

Table 2 presents a comparison of the outcome measures for the intervention and control groups. A repeated two-way analysis of variance revealed a significant group (intervention vs. control) × time (baseline vs. follow-up) interaction for COPM performance (F = 4.021, *p* = 0.048) and satisfaction (F = 3.583, *p* = 0.049). Subsequent multiple comparisons (Figure 3) identified significant differences in both COPM performance and satisfaction (*p* < 0.05). Regarding effect size, COPM performance (Cohen’s *d* = 0.93) demonstrated a large effect, while COPM satisfaction (Cohen’s *d* = 0.73) showed a medium effect. Moreover, the within-group comparisons revealed that the intervention group’s mean COPM performance score increased significantly from 3.2 ± 2.0 at baseline to 7.6 ± 1.7 at follow-up. In a previous study, the MCIDs for COPM performance and satisfaction were reported as 2.20 (95% CI: 1.80–2.59) and 2.06 (95% CI: 1.73–2.39), respectively [21]. The improvements observed in this study exceeded these MCIDs, indicating a clinically meaningful improvement in addition to statistical significance. However, although the NRS showed a significant interaction effect (F = 5.778, *p* = 0.019), the subsequent multiple comparisons were not significant (Figure 3), and its small effect size suggests that the intervention may not have had a pronounced impact on pain levels as measured by the NRS. Furthermore, for HADS (anxiety and depression), EQ-5D (quality of life), and PDAS (physical functioning), no statistically significant differences were found, although both the intervention and control groups showed a trend toward improvement.

### 3.3. Results of Early-Postoperative Coping Strategies

#### 3.3.1. Overview of Coping Strategies

During the second to fourth postoperative weeks, the intervention group (n = 14) demonstrated a mean acquisition of 15.07 effective coping strategies (SD = 6.68). A total of 210 effective coping strategies were identified across the 14 patients.

#### 3.3.2. Categorization

**Figure 4:**
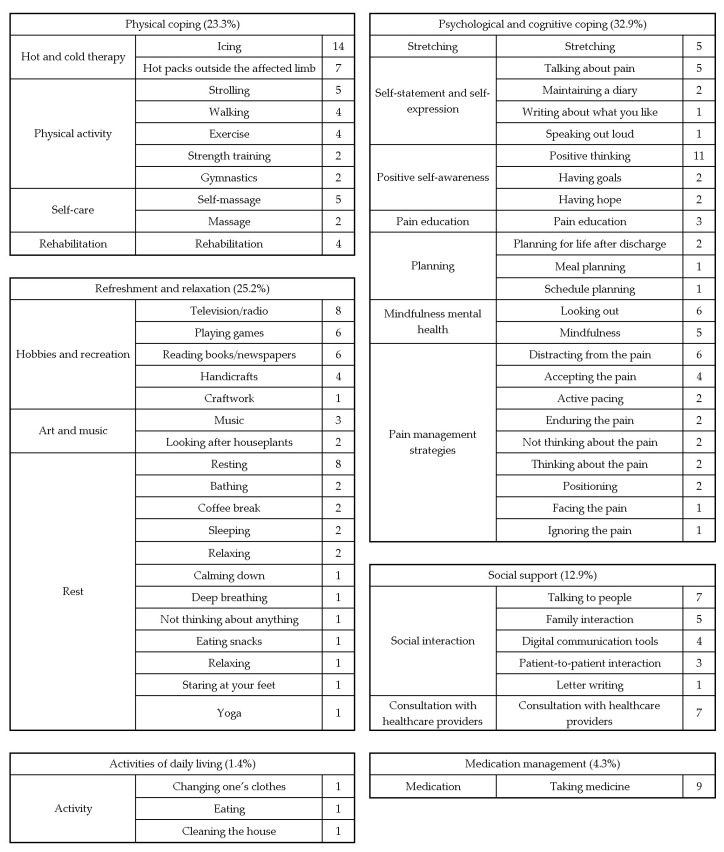
Pain management strategies: an affinity diagram.

Using the KJ method, the strategies were grouped into eighteen categories and further consolidated into six overarching groups (Figure 4).

Regarding physical coping (23.3%), the focus was on managing inflammation and restoring function. The purpose was to enhance physical comfort and promote postoperative functional recovery.

Regarding psychological and cognitive coping (32.9%), the focus was on stress management, meditation, and positive thinking. The purpose was cognitive pain management, reducing anxiety, and fostering positive thought patterns.

Regarding social support (12.9%), the focus was on emotional, informational, and practical support from family, friends, healthcare professionals, and peers. The purpose was to provide reassurance and resources for managing postoperative pain and anxiety.

Regarding refreshment and relaxation (25.2%), the focus was on hobbies, music, reading, and rest. The purpose was to alleviate physical/mental tension, offer distraction, and improve overall well-being.

Regarding activities of daily living (1.4%), the focus was on strategies to support independence in daily tasks.

Regarding medication management (4.9%), the focus was on pain management through analgesics and other medications.

### 3.4. Key Observations

Psychological and cognitive coping (32.9%) and refreshment and relaxation (25.2%) were the most frequently employed strategies. All 14 patients used strategies in the domains of physical coping, psychological and cognitive coping, social support, and refreshment and relaxation. Three patients adopted activities of daily living strategies, and nine used medication management strategies.

### 3.5. Analysis and Data Review

After the study ended, all coping strategy data were analyzed using the KJ method to determine group relationships and patterns. Detailed categorizations and proportions are available in Table 3.

## 4. Discussion

This study aimed to evaluate the impact of OT employing coping strategies on goal achievement, pain, psychological factors, and QOL in early-postoperative patients who had undergone TKA. Additionally, a qualitative analysis of the effective coping strategies for managing pain and anxiety as reported by the patients in the intervention group was conducted to characterize coping strategies in the early-postoperative period.

The significance of this study lies in it being the first to examine the effectiveness of OT combined with coping strategies for early-postoperative pain management in patients undergoing TKA. The results showed that the intervention group’s COPM scores (performance and satisfaction) improved, and the changes remained statistically significant. These results suggest that patients’ understanding of pain management and active use of pain coping techniques resulted in improved self-efficacy and goal achievement [33]. Therefore, facilitating activities through OT can enhance self-efficacy and support goal achievement [12]. Additionally, Riddle et al. [34] have demonstrated that temporary pain management, self-directed relaxation, and self-directed activity control skills in patients with knee osteoarthritis improved life functioning. These findings suggest that incorporating coping techniques into OT for patients undergoing TKA may facilitate goal achievement.

Furthermore, the statistically significant pain-relieving effects of coping strategies on the NRS scores in both groups support the results of previous studies. For example, Wilson et al. [35] showed that patients learning specific coping strategies for pain through an online self-management program experienced improved pain self-efficacy and effective pain relief. Similarly, Kemp et al. [36] demonstrated that coping strategies routinely adopted by older adults, such as exercise, cold therapy, and prayer, improved pain perception. These findings align with our results and underscore the importance of patients learning specific strategies to cope with pain during the postoperative period.

Early-postoperative coping strategies were categorized into six groups: physical coping, psychological and cognitive coping, social support, refreshment and relaxation, activities of daily living, and medication management. Prior studies have identified analgesics (78%), exercise (35%), and cognitive methods (37%) as the most commonly used coping strategies for chronic pain [37]. Other studies have reported the use of medication (73%), exercise (33%), and cognitive strategies, including distraction (31%) [38]. Recent studies have highlighted the process by which different attitudes and beliefs lead to changes in daily life and activities when coping with chronic pain, noting that this process requires self-involvement and is influenced by demographic characteristics, physical factors, psychosocial factors, and self-knowledge about one’s condition and coping strategies [16]. Furthermore, coping strategies for patients with high preoperative anxiety include conversations with healthcare professionals [39]. The current study also included these elements as characteristics of early-postoperative coping strategies (Table 3).

However, psychological and cognitive coping strategies were the most prevalent in this study, highlighting a limitation in the current provision of CBT in Asian countries, including Japan. The foundation for delivering CBT remains underdeveloped, posing challenges to implementing psychological approaches [40]. Based on these findings, we emphasize the importance of OT that integrates coping strategies for managing pain and psychological factors in the early-postoperative period. Furthermore, the importance of comprehensive interventions tailored to individual conditions and characteristics should be established as early-postoperative coping strategies—an underexplored area requiring further study. We did not identify significant differences in psychological factors, life function, and QOL. This suggests that, while the intervention positively affected occupational performance and pain reduction, it may not have directly influenced other factors. However, a previous study conducted with patients undergoing knee replacement surgery and experiencing moderate-to-severe pain has reported no additional benefit from pain coping strategy training compared with usual care or arthritis education [41]. While some studies have reported substantial improvements in patients with knee osteoarthritis when comparing a CBT group with a standard-care group [42,43], our intervention comprised an OT-based coping strategy approach consistent with Hara et al. [15]. While a more effective OT-based coping strategy intervention may require additional CBT, these factors were not verified in the present study. Therefore, combining OT intervention with CBT may enhance effectiveness and warrants further investigation.

### Limitations

There are several important limitations to this study. First, the study was limited to female patients, restricting the generalizability of the results. Future research should include a more diverse sample to improve applicability. Second, participants were not randomly assigned to treatment options, making it unclear whether observed differences were due to the intervention or pre-existing group differences. To address this, randomized controlled trials (RCTs) or propensity score matching (PSM) should be considered. Third, the inability to blind treatment options and reliance on qualitative data may have introduced bias. Future studies should implement evaluator blinding where possible and incorporate objective outcome measures. Fourth, the use of G*Power with a power of 0.5 may have limited the ability to detect effects. This study was an early-stage exploratory investigation conducted with limited resources and time; future research should strive for higher power levels (e.g., 0.8 or 0.9) and larger sample sizes to enhance statistical reliability. Finally, the study periods differed between groups, raising the possibility of external influences. The control and intervention procedures were conducted in different years (January to December 2021 and January to December 2022, respectively), which may have introduced time-related confounding factors. To mitigate this, future studies should ensure simultaneous data collection or apply statistical adjustments such as ANCOVA. By addressing these limitations through randomization, blinding, increased power, and synchronized data collection, future studies can strengthen the reliability and applicability of findings.

## 5. Conclusions

While the intervention had a statistically significant positive effect on occupational performance and satisfaction (*p* < 0.05), it may not have directly influenced other factors such as psychological well-being or QOL. The qualitative analysis identified 210 effective coping strategies and categorized them into six main themes: physical coping, psychological and cognitive coping, social support, refreshment and relaxation, activities of daily living, and medication management. The prevalence of psychological and cognitive coping (32.9%) and refreshment and relaxation (25.2%) highlights the diverse nature of effective postoperative coping mechanisms. These findings suggest that early intervention should incorporate psychological, social, and relaxation techniques in addition to physical rehabilitation. By addressing both physical and psychosocial aspects, healthcare providers can better assist patients in achieving their postoperative goals and enhancing their overall well-being.

## Figures and Tables

**Table 1:** Characteristics of the participants.

	Total (N = 28)	Intervention Group (n = 14)	Control Group (n = 14)	*p*-Value
Demographic information				
Sex [female, (%)]	28 (100)	14 (100)	14 (100)	1.00
Age (years)	76.8 (6.0)	75.7 (5.7)	77.9 (6.0)	0.34
BMI (kg/m^2^)	27.4 (5.1)	27.5 (4.7)	27.3 (5.4)	0.93
Worker (%)	7 (25.0)	5 (35.7)	2 (14.3)	0.20
Smoker (%)	1 (3.6)	0 (0.0)	1 (7.1)	0.33
Drinker (%)	2 (7.1)	1 (7.1)	1 (7.1)	1.00
KL grade [n (%)]				1.00
III	6 (21.4)	3 (10.7)	3 (10.7)	
IV	22 (78.6)	11 (39.3)	11 (39.3)	
Long-term care insurance				0.57
No care (%)	23 (82.1)	11 (78.6)	12 (85.7)	
Required support I (%)	2 (7.1)	1 (3.6)	1 (3.6)	
Required support II (%)	3 (10.7)	2 (14.3)	1 (7.1)	

*Note.* Values indicate mean (SD) or number (%). BMI, body mass index; and KL grade, Kellgren–Lawrence grade.

**Table 2:** Baseline and follow-up outcomes.

	Intervention Group (n = 14)		Control Group (n = 14)		Time-by-Group Interaction		Effect Size
	Baseline	Follow-Up	Baseline	Follow-Up	F-Value	*p*-Value	Cohen’s *d*
COPM performance	3.2 ± 2.0	7.6 ± 1.7 *	3.4 ± 1.9	5.5 ± 2.6	4.021	0.048	0.93
COPM satisfaction	2.7 ± 1.9	7.9 ± 2.0 *	3.4 ± 2.4	5.6 ± 2.8	3.583	0.049	0.73
NRS	5.0 ± 1.7	1.5 ± 1.1	3.2 ± 2.0	1.6 ± 1.0	5.778	0.019	0.13
HADS anxiety	6.4 ± 3.1	1.8 ± 1.6	4.6 ± 3.7	2.4 ± 3.1	2.427	0.125	0.26
HADS depression	6.5 ± 3.0	2.9 ± 1.7	6.4 ± 3.5	3.2 ± 3.2	0.099	0.754	0.17
EQ-5D	0.589 ± 0.225	0.844 ± 0.113	0.630 ± 0.122	0.784 ± 0.140	1.349	0.250	0.45
PDAS	27.1 ± 14.5	11.0 ± 9.7	27.5 ± 8.6	16.5 ± 9.3	0.764	0.386	0.57

*Note.* Values are expressed as mean ± standard deviation. COPM, Canadian Occupational Performance Measure; NRS, Numeric Rating Scale; HADS, Hospital Anxiety and Depression Scale; EQ-5D-5L, EuroQol 5-Dimension 5-Level; and PDAS, Pain Disability Assessment Scale. * Significant difference in post-occupational therapy results between the groups (*p* < 0.05).

**Table 3:** Percentage of each coping strategy implemented among early-postoperative in-patients.

Physical coping	14 (100%)
Psychological and cognitive coping	14 (100%)
Social support	14 (100%)
Refreshment and relaxation	14 (100%)
Activities of daily living	3 (21.4%)
Medication management	9 (64.3%)

## Data Availability

The clinical trial registration information for this research is available online. The documents can be viewed at UMIN (ID number: UMIN000050536).

## Abbreviations

The following abbreviations are used in this manuscript:

TKA	Total knee arthroplasty
QOL	Quality of life
CBT	Cognitive behavioral therapy
OT	Occupational therapy
COPM	Canadian Occupational Performance Measure
NRS	Numeric Rating Scale
EQ-5D-5L	EuroQol 5-Dimension 5-Level
HADS	Hospital Anxiety and Depression Scale
PDAS	Pain Disability Assessment Scale
KJ	Jiro Kawakita
BMI	Body mass index
KL	Kellgren–Lawrence grade
